# Variations in Age-Adjusted Prostate Cancer Incidence Rates by Race and Ethnicity After Changes in Prostate-Specific Antigen Screening Recommendation

**DOI:** 10.1001/jamanetworkopen.2022.40657

**Published:** 2022-11-07

**Authors:** Sue-Min Lai, John Keighley, Sarma Garimella, Mollee Enko, William P. Parker

**Affiliations:** 1 Kansas Cancer Registry, Department of Population Health, University of Kansas Medical Center, Kansas City; 2 Department of Orthopedic Surgery and Sports Medicine, University of Kansas Medical Center, Kansas City; 3 Department of Biostatistics and Data Science, University of Kansas Medical Center, Kansas City; 4 Department of Urology, University of Kansas Medical Center, Kansas City

## Abstract

**Importance:**

After publication of US Preventive Task Force Prostate-Specific Antigen (PSA) screening guidelines in 2008 and 2012, there have been documented associations with incidence and stage distributions of prostate cancer. It is unclear if these changes were temporary or differed by age or race and ethnicity.

**Objective:**

To assess the association of 2008 and 2012 PSA guidelines with prostate cancer incidence by age and race and ethnicity in the US.

**Design, Setting, and Participants:**

This cross-sectional study evaluated prostate cancer incidence from 2005 to 2018 in the US using data from the US Cancer Statistics public use database. Data were analyzed from August 2020 through June 2022.

**Main Outcomes and Measures:**

The primary outcome was the year when rates of prostate cancer incidence changed directionality by age and race and ethnicity. Age-adjusted incidence rates of prostate cancer and corresponding 95% CIs were created, followed by join point regression analysis to evaluate trends of age-adjusted incidence rates of prostate cancer by age, race, Hispanic ethnicity, and stage of diagnosis.

**Results:**

Among 2 944 387 men with prostate cancer, 2 869 943 (97.5%) men were aged 50 years and older. Men aged 50 years and older accounted for 185 476 of 191 533 Hispanic individuals (96.8%) and 2 684 467 of 2 752 854 non-Hispanic individuals (97.5%). Men aged 50 years and older accounted for 427 016 of 447 847 African American individuals (95.4%), 12 141 of 12 470 American Indian or Alaska Native individuals (97.4%), 61 126 of 62 159 Asian or Pacific Islander individuals (98.3%), and 2 294 171 of 2 344 392 White individuals (97.9%). Men with unknown race (77 519 men) were excluded from the analysis. A decrease in age-adjusted rate of prostate cancer after the 2008 guideline change was observed in all age groups by race and ethnicity. For example, among African American men ages 65 to 74 years, 10 784 of 807 080 men (1.34%) had a prostate cancer diagnosis in 2007 vs 10 714 of 835 548 men in 2008 (1.28%). The mean annual age-adjusted incidence rates of prostate cancer per 100 000 men were 157.7 men (95% CI, 157.4-158.0 men) in 2005 to 2008 and 131.9 men (95% CI, 131.6-132.2 men) in 2009 to 2012. The number of inflections and annual percent changes (APCs) for segments separated by inflections varied by age, race, and Hispanic ethnicity. For men ages 65 to 74 years, the APC was −6.53 (95% CI, −9.28 to −3.69) for 2009 to 2014 among African American men (2 join points), −5.96 (95% CI, −6.84 to −5.07) for 2007 to 2018 among American Indian or Alaska Native men (1 join point), −6.52 (95% CI, −9.22 to −3.74) for 2007 to 2014 among Asian or Pacific Islander men (2 join points), −7.92 (95% CI, −11.36 to −4.35) for 2009 to 2014 among Hispanic men (2 join points), and −7.02 (95% CI, −9.41 to −4.57) for 2007 to 2014 among White men (2 join points).‬‬‬‬‬‬‬‬

**Conclusions and Relevance:**

In this study, men in different age, race, and ethnicity groups had different APC patterns after 2008 and 2012 PSA screening guideline changes. These findings may provide important data on the timing and durations of changes in cancer diagnoses that are associated with changes in PSA screening recommendations and may be valuable for targeted strategies to reduce regional- and distant-staged cancers.

## Key Points

**Question:**

Were changes in prostate-specific antigen (PSA) screening guidelines in 2008 and 2012 associated with incidence and stage distribution of prostate cancers by age and race and ethnicity in the US?

**Findings:**

This cross-sectional study of 2 944 387 men with prostate cancer found that 2008 and 2012 US Preventive Services Task Force recommendations were associated with different changes in prostate cancer incidence among African American, American Indian or Alaska Native, Asian Pacific and Islander, Hispanic, and White populations. The year when age-adjusted rates of prostate cancer reversed direction from 1 period to the next differed by group.

**Meaning:**

These findings suggest that when recommendations are made, strategies should be developed to target men of different age and race and ethnicity groups to ensure equitable health care.

## Teaser

This cross-sectional study investigates the association of changes in prostate-specific antigen screening guidelines with changes in incidence of prostate cancer by race and ethnicity.

## Introduction

Benefits associated with cancer screening have been extensively documented, in particular, the association of early detection with improved survival in breast and cervical cancers.^1^ Prostate-specific antigen (PSA) screening, however, has generated a significant debate concerning questionable survival benefits and competing associations with quality of life among men after treatment, such as surgery and radiation therapy.^2,3^ In 2012, the US Preventive Services Task Force (USPSTF) recommended against PSA-based screening for prostate cancer (grade D).^4^ Decreased diagnosis of prostate cancers after the 2012 revised PSA screening guidelines was anticipated and has been reported.^5,6,7^ Previous investigators^6,7^ focused on temporal trends in prostate cancer incidence, decreased PSA screenings, decreased reported incidence rates, and increased late-stage burden. Source of data, population coverage, years of diagnosis, and analytic approach varied. One study^5^ calculated a series of percentage decreases between 2 consecutive years of age-adjusted rates to identify which segments should be further evaluated. Other studies^7,8,9^ captured annual percent change (APC) of age-adjusted rates to evaluate prostate cancer incidence trends using data from the Surveillance, Epidemiology, and End Results (SEER) program. Most of these studies included prostate cancers diagnosed in earlier years (eg, 1995 to 2012 and 2004 to 2014). All but 1 study used data from the SEER program, which had coverage of 9, 13, 18, or 21 geographic areas in the US (equivalent to 18% to 33% of the US population).^5,6,7,8^ The Annual Report to the Nation on the Status of Cancer^10^ was based on 42 state central registries covering 89% of the US population, but it was limited to diagnoses from 2001 to 2014 in Black and White men. American Indian or Alaska Native, Asian or Pacific Islander, and Hispanic individuals were not included in these studies. PSA screening based on survey was used to examine its association with decreased rates of prostate cancer.^8,10^ The association observed between PSA screening based on survey and trends in age-adjusted prostate cancer incidence rates may be misleading because state- and national-level survey data were not specific to men diagnosed with prostate cancer. Furthermore, the survey question asked if a PSA test was done in the prior year. The response would be “no” for men with interval screenings.

Racial disparities in prostate cancer incidence have been found mostly between African American and White men in the US but not in American Indian or Alaska Native, Asian or Pacific Islander, or Hispanic individuals vs White individuals; this was also the case for decreasing prostate cancer incidence after PSA guideline changes.^5,6,7,9^ It is currently unknown if or how long the decrease would continue or the extent of the decrease, particularly in American Indian or Alaska Native, Asian or Pacific Islander, and Hispanic populations. The goal of this study was to include all prostate cancers diagnosed between 2005 and 2018 in the US and examine the association of race, Hispanic ethnicity, and stage with changes in prostate cancer incidence rates after the 2008 and 2012 USPSTF PSA screening guidelines, including the timing and duration of these changes.^4,11^ We hypothesized that patterns would differ among 5 race and ethnicity groups (African American, American Indian or Alaska Native, Asian or Pacific Islander, Hispanic, and White populations).

## Methods

The institutional review board of the University of Kansas Medical Center determined that this cross-sectional study was exempt from review and informed consent because it used deidentified public-use data. Our study followed the Strengthening the Reporting of Observational Studies in Epidemiology (STROBE) reporting guideline for cross-sectional studies.

### Data Sources

This study used the currently available prostate cancer incidence data in the United States through the US Cancer Statistics (USCS) public use database, which covers 50 states and the District of Columbia. The public use data set contains deidentified cancer incidence data from the Centers for Disease Control and Prevention National Program of Cancer Registries (NPCR) and National Cancer Institute SEER programs.^12^ Data items extracted from the USCS public use database were demographics (age, race, and Hispanic ethnicity) and tumor characteristics (year of diagnosis, histology, and stage at diagnosis).

### Study Design

We identified men with invasive prostate cancers that were consistent with the *International Classification of Diseases for Oncology, Third Revision* (*ICD-O-3*) topography code C61.9 and *ICD-O-3* behavior code 3 (SEER site recode 28010) and were diagnosed in 2005 to 2018 to account for 2008 and 2012 PSA guideline recommendation changes. Data items included in the analysis were demographics (age in 5-year groups, race, and Hispanic ethnicity) and tumor characteristics (year of diagnosis, histology, and stage at diagnosis). Age was grouped into younger than 65 years, ages 65 to 74 years, and ages 75 years and older at diagnosis. Race was classified according to NPCR and SEER as African American, American Indian or Alaska Native, Asian or Pacific Islander, and White. Race and ethnicity were self-reported. Race and Hispanic ethnicity were mostly based on medical records and reported to state cancer registries, with 2 modifications: American Indian or Alaska Native was validated via linkages between the Indian Health Service and state cancer registries to ascertain American Indian or Alaska Native status for individuals with mixed race.^13^ Hispanic ethnicity was validated using the Hispanic Identification Algorithm version 2, which was developed by the North America Association Central Cancer Registries.^14^ After we applied the algorithm, individuals not classified as Hispanic were classified as non-Hispanic, leaving no individuals with unknown Hispanic ethnicity status. Stage at diagnosis was classified as localized, regional, distant, and unknown according to the SEER derived summary staging 2000 for diagnoses from 2005 to 2015 based on collaborative staging variables (eg, tumor size, extension, number of lymph nodes positive and examined, and metastasis at diagnosis), prostate cancer site-specific factors (eg, PSA laboratory value), and SEER summary stage 2000 for cancers diagnosed from 2016 to 2017 and directly assigned summary stage 2018 for individuals diagnosed in 2018. Individuals with unknown race or ethnicity and cancers with unknown stage information were not included in the analysis.

### Statistical Analysis

Cancer counts, age-adjusted incidence rates, and corresponding 95% CIs were created using SEER*Stat statistical software version 8.3.9 (SEER).^15^ The age-adjusted incidence rate was the number of incident prostate cancers per 100 000 men and was age adjusted to the 2000 US standard population.^16^ Race and ethnicity were treated as separate measures. Joinpoint Regression Program software version 4.9.0.0 (National Cancer Institute Surveillance Research Program) identifies trends by fitting a model including 2 or more linear segments that have different slopes at the time or calendar year when the change in trend occurred.^17,18^ For the number of join points (or inflection points) identified, we started with the minimum number of join points (eg, 0 join points) and tested if more join points were statistically significant, adding these using the grid search approach.^19^ A regression line with 2 join points implies 3 line segments with 3 different slopes of annual incidence rates over time. APCs, or slopes corresponding to each join point segment, and associated *P* values were reported by age, race, Hispanic ethnicity, and stage at diagnosis combinations. A 2-sided *P* value ≤ .05 was considered statistically significant. Data were analyzed from August 2020 through June 2022.

## Results

Among 2 944 387 men with primary prostate cancer that was diagnosed from 2005 to 2018, 2 869 943 (97.5%) men were aged 50 years and older. Men aged 50 years and older accounted for 185 476 of 191 533 Hispanic individuals (96.8%) and 2 684 467 of 2 752 854 non-Hispanic individuals (97.5%). Men aged 50 years and older accounted for 427 016 of 447 847 African American individuals (95.4%), 12 141 of 12 470 American Indian or Alaska Native individuals (97.4%), 61 126 of 62 159 Asian or Pacific Islander individuals (98.3%), and 2 294 171 of 2 344 392 White individuals (97.9%). Men with unknown race (77 519 men) were excluded from the analysis. Prostate cancer counts and US male population by year of diagnosis, age group, race, and Hispanic ethnicity are provided in eTables 1 and 2 in the Supplement. A decrease in age-adjusted rate of prostate cancer after the 2008 guideline change was observed in all age groups by race and ethnicity. For example, among African American men ages 65 to 74 years, 10 784 of 807 080 men (1.34%) had a prostate cancer diagnosis in 2007 vs 10 714 of 835 548 men in 2008 (1.28%). The US mean annual age-adjusted incidence rates of prostate cancer per 100 000 men corresponding to 2008 and 2012 changes in the USPSTF recommended prostate cancer screening were 157.7 men (95% CI, 157.4-158.0 men) in 2005 to 2008, 131.9 men (95% CI, 131.6-132.2 men) in 2009 to 2012, and 106.4 men (95% CI, 106.2-106.6 men) in 2013 to 2018.‬‬‬‬‬‬

The number of inflection points and APCs for line segments separated by inflection points varied by age group stratified by race (Table 1) and by Hispanic ethnicity (eTable 3 in the Supplement). For example, the APC for ages 65 to 74 years was −6.53 (95% CI, −9.28 to −3.69; *P* = .001) in African American men (who had 2 join points) from 2009 to 2014, −5.96 (95% CI, −6.84 to −5.07; *P* < .001) among American Indian or Alaska Native men (who had 1 join point) from 2007 to 2018, −6.52 (95% CI, −9.22 to −3.74; *P* = .001) from 2007 to 2014 for Asian or Pacific Islander men (who had 2 join points), and −7.02 (95% CI, −9.41 to −4.57;*P* < .001) among White men (who had 2 join points) from 2007 to 2014 (Table 1). The APC for Hispanic men ages 65 to 74 years (who had 2 join points) was −7.92 (95% CI, −11.36 to −4.35; *P* = .002) from 2009 to 2014 (eTable 3 in the Supplement). APCs for other age groups by race are in Table 1. APCs for Hispanic men aged younger than 65 years and 75 years and older are in eTable 3 in the Supplement.

**Table 1.** APC in Age-Adjusted Prostate Cancer Incidence by Age and Race

Age group, y	Join points, No.	Time segment	APC (95% CI)	*P* value
**African American**				
<65	2	2005-2009	1.98 (−1.51 to 5.60)	.22
		2009-2013	−6.21 (−11.24 to −0.90)	.03
		2013-2018	−0.69 (−3.10 to 1.79)	.52
65-74	2	2005-2009	0.18 (−2.77 to 3.22)	.89
		2009-2014	−6.53 (−9.28 to −3.69)	.001
		2014-2018	0.66 (−2.30 to 3.72)	.61
≥75	2	2005-2007	−0.21 (−9.93 to 10.56)	.96
		2007-2014	−7.92 (−9.50 to −6.31)	<.001
		2014-2018	−0.97 (−4.13 to 2.29)	.49
**American Indian or Alaska Native**				
<65	0	2005-2018	−4.18 (−5.14 to −3.21)	<.001
65-74	1	2005-2007	3.83 (−9.71 to 19.41)	.56
		2007-2018	−5.96 (−6.84 to −5.07)	<.001
≥75	0	2005-2018	−6.66 (−8.15 to −5.16)	<.001
**Asian or Pacific Islander**				
<65	2	2005-2008	5.69 (−2.23 to 14.26)	.13
		2008-2014	−6.53 (−9.74 to −3.22)	.003
		2014-2018	0.59 (−4.25 to 5.67)	.78
65-74	2	2005-2007	4.82 (−11.88 to 24.67)	.53
		2007-2014	−6.52 (−9.22 to −3.74)	.001
		2014-2018	1.93 (−3.51 to 7.68)	.43
≥75	1	2005-2014	−8.32 (−9.99 to −6.63)	<.001
		2014-2018	2.65 (−3.66 to 9.39)	.38
**White**				
<65	2	2005-2007	8.88 (−5.76 to 25.80)	.20
		2007-2014	−6.73 (−8.98 to −4.43)	<.001
		2014-2018	0.12 (−4.35 to 4.79)	.95
65-74	2	2005-2007	5.38 (−9.65 to 22.92)	.44
		2007-2014	−7.02 (−9.41 to −4.57)	<.001
		2014-2018	2.41 (−2.46 to 7.52)	.28
≥75	2	2005-2007	0.28 (−11.73 to 13.93)	.10
		2007-2013	−9.11 (−11.67 to −6.48)	<.001
		2013-2018	1.29 (−1.56 to 4.22)	.31

Abbreviation: APC, annual percent change.

**Figure 1.**
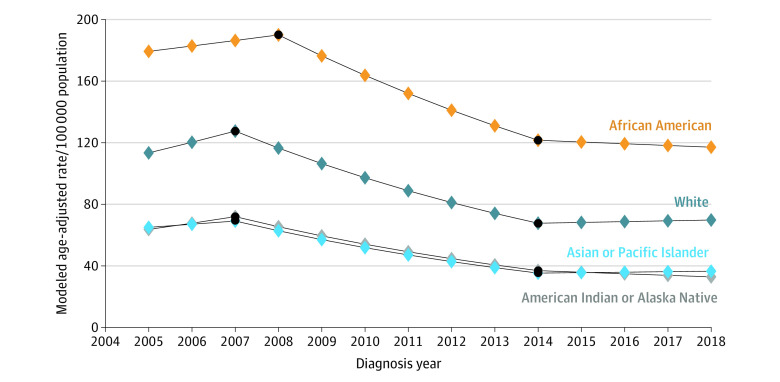
Join Point Trend Analysis of Localized Prostate Cancers by Race Black dots indicate join point locations.

**Figure 2.**
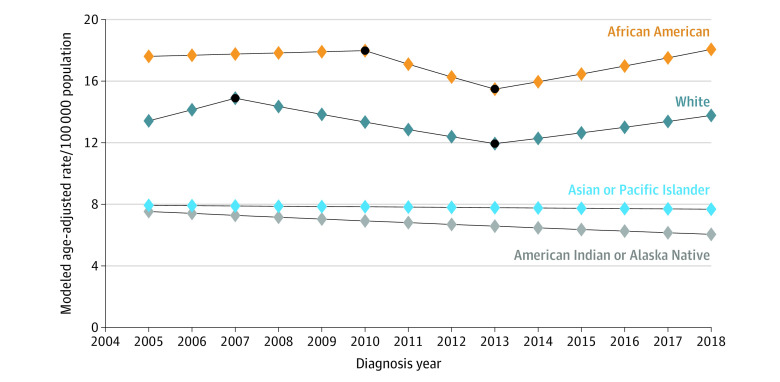
Join Point Trend Analysis of Regional Prostate Cancers by Race Black dots indicate join point locations.

**Figure 3.**
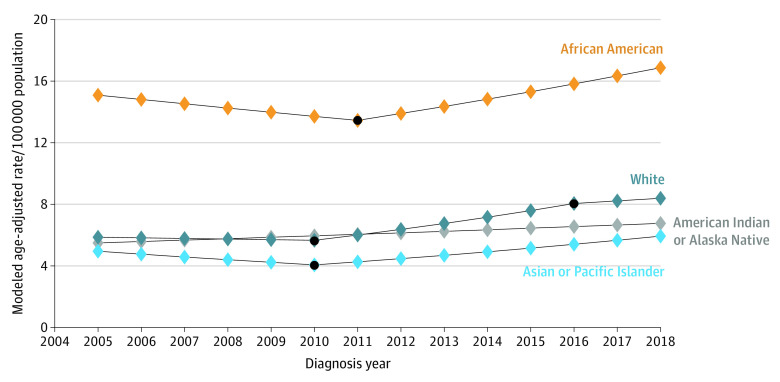
Join Point Trend Analysis of Distant Prostate Cancers by Race Black dots indicate join point locations.

Figure 1, Figure 2, and Figure 3 show join point trend analyses of annual age-adjusted incidence of localized, regional, and distant stage cancer, respectively, stratified by race. These outcomes are shown for Hispanic and non-Hispanic ethnicity in eFigure 1 to eFigure 3 in the Supplement. The number of join points and APCs stratified by stage at diagnosis, race, and Hispanic ethnicity are shown in Table 2 and eTable 4 in the Supplement. African American and Hispanic men with localized prostate cancer had 2 inflections and 3 APCs, with significant decreases in age-adjusted rates between 2008 and 2014 (African American: APC, −7.17 [95% CI, −9.48 to −4.80]; *P* < .001; Hispanic: APC, −8.80 [95% CI, −10.69 to −6.87]; *P* < .001). There were also 2 inflections, with the first inflection occurring in 2007 or 1 year before the PSA recommendation was published, for American Indian or Alaska Native (APC, −9.10 [95% CI, −12.09 to −6.01; *P* < .001), Asian or Pacific Islander (APC, −9.16 [95% CI, −12.36 to −5.84]; *P* = .001), and White (APC, −8.65 [95% CI, −11.07 to −6.17; *P* < .001) men. Details on year of inflection and APC for all study groups are in Table 2 and eTable 4 in the Supplement.

**Table 2.** APC in Age-Adjusted Prostate Cancer Incidence by Stage at Diagnosis and Race

Stage at diagnosis^a^	Join points	Time segment	APC (95% CI)	*P* value
Localized				
African American	2	2005-2008	1.95 (−3.63 to 7.86)	.43
		2008-2014	−7.17 (−9.48 to −4.80)	<.001
		2014-2018	−0.94 (−4.41 to 2.65)	.54
American Indian or Alaska Native	2	2005-2007	6.31 (−12.78 to 29.57)	.48
		2007-2014	−9.10 (−12.09 to −6.01)	<.001
		2014-2018	−2.85 (−8.74 to 3.42)	.30
Asian or Pacific Islander	2	2005-2007	3.11 (−16.59 to 27.48)	.74
		2007-2014	−9.16 (−12.36 to −5.84)	.001
		2014-2018	0.87 (−5.67 to 7.87)	.76
White	2	2005-2007	6.05 (−9.51 to 24.29)	.40
		2007-2014	−8.65 (−11.07 to −6.17)	<.001
		2014-2018	0.8 (−4.16 to 5.95)	.72
Regional				
African American	2	2005-2010	0.41 (−2.36 to 3.27)	.73
		2010-2013	−4.87 (−16.08 to 7.83)	.37
		2013-2018	3.14 (0.29 to 6.07)	.04
American Indian or Alaska Native	0	2005-2018	−1.67 (−3.39 to 0.07)	.06
Asian or Pacific Islander	0	2005-2018	−0.25 (−1.60 to 1.13)	.70
White	2	2005-2007	5.34 (−8.62 to 21.44)	.41
		2007-2013	−3.62 (−6.63 to −0.50)	.03
		2013-2018	2.89 (−0.33 to 6.21)	.07
Distant				
African American	1	2005-2011	−1.90 (−3.52 to −0.24)	.03
		2011-2018	3.29 (1.93 to 4.66)	<.001
American Indian or Alaska Native	0	2005-2018	1.61 (−0.28 to 3.53)	.09
Asian or Pacific Islander	1	2005-2010	−3.85 (−7.17 to −0.42)	.03
		2010-2018	4.84 (3.06 to 6.65)	<.001
White	2	2005-2010	−0.69 (−1.57 to 0.19)	.11
		2010-2016	6.04 (5.11 to 7.00)	<.001
		2016-2018	2.10 (−1.87 to 6.22)	.25

Abbreviation: APC, annual percent change.

^a^ Surveillance, Epidemiology, and End Results summary stage at diagnosis.

Results from join point trend analysis of regional stage prostate cancer are shown in Figure 2 by race and eFigure 2 in the Supplement by Hispanic ethnicity. Among American Indian or Alaska Native and Asian or Pacific Islander men, there was no inflection throughout 2005 to 2018 (Table 2). Hispanic men had no inflection either, but the age-adjusted regional stage cancer continued to decrease from 2005 to 2018 (APC, −1.07 [95% CI, −1.76 to −0.37]; *P* = .006) (eTable 4 in the Supplement). African American men had an increase in regional stage cancer from 2013 to 2018 (APC, 3.14 [95% CI, 0.29 to 6.07]; *P* = .04), while White men experienced a decrease in regional stage cancer from 2007 to 2013 (APC, −3.62 [95% CI, −6.63 to −0.50]; *P* = .03), followed by no significant change (APC, 2.89 [95% CI, −0.33 to 6.21]; *P* = .07) from 2013 to 2018.

The pattern of annual age-adjusted rates of distant stage prostate cancers was also dynamic (Table 2; eTable 4 in the Supplement). American Indian or Alaska Native men had no significant change from 2005 to 2018. African American and Asian or Pacific Islander men had 1 inflection (African American men in 2011 and Asian or Pacific Islander men in 2010) (Table 2). Hispanic and White men had 2 inflections, with the segment between the first and second inflections showing a significant increase in age-adjusted rates (Hispanic: APC 4.60; [95% CI, 1.36-7.94]; *P* = .01 from 2011 to 2016; White: APC, 6.04 [95% CI, 5.11-7.00]; *P* < .001 from 2010 to 2016).

## Discussion

In this cross-sectional study of prostate cancer incidence from 2005 to 2018, an overall decrease in age-adjusted rates after the 2008 guideline change was observed in all 3 age groups and by race and ethnicity and stage at diagnosis. The number of inflection points and APCs for time segments separated by inflections were different by age group, race, and Hispanic ethnicity. The year of inflection in age-adjusted rates also differed by race and Hispanic ethnicity.

Health guidelines have long been used to direct the management of many health issues and have the potential to influence health at a population level. As found in this and in previous analyses,^20,21,22,23^ even well-intentioned guidelines may be associated with negative outcomes with respect to the epidemiology of diseases. In our study, changes in the incidence of prostate cancer were found after the 2008 and 2012 PSA screening recommendations. In prior analyses, in addition to finding reductions in incidence (likely associated with decreased PSA screening), researchers were able to identify how changes in the incidence of prostate cancer after guideline recommendation changes differed by age, between African American and White individuals,^6,7,8,10^ and by ethnic group.^8^ Using join point regression, we were able to identify differences by race and ethnicity in when age-adjusted rates of prostate cancer started to decrease relative to guideline publication. We were also able to characterize the associated rates of change specific to those groups and the year when rates of prostate cancer began to increase.

Our study analyzed 3 time periods that corresponded to cancer diagnoses prior to the 2008 revised USPSTF recommendation, after 2008 and before the 2012 revised recommendation, and after the 2012 revised recommendation. A decrease in age-adjusted rates was found for all 3 age groups (ages <65, 65-74, and ≥75 years) at diagnosis regardless of race (Table 1), similar to data evaluating the association specifically of the 2012 recommendation with rates of cancer diagnosis.^21,22,23^ However, the timeline of significant decreases in age-adjusted rates varied by race, ethnicity, and age group. For example, among men aged 65 to 74 years, the rate of newly diagnosed prostate cancer decreased at approximately 7% per year beginning in 2007 for White men, while a decrease did not occur until 2009 among African American and Hispanic men. Furthermore, we found that within stages of prostate cancer, incidence similarly varied by race and ethnicity and age, supporting previous work^24^ finding that the USPSTF recommendation was associated with an apparent stage migration toward higher-risk disease in men who were diagnosed.

Understanding the temporal nature of the association of guidelines with clinical practice is important for many reasons. First this may provide an understanding of the rate of adoption of guidelines in the community setting. Given that prostate cancer is a disease whose management begins in most instances with a primary care physician electing to initiate screening, the incidence of prostate cancer can be considered a reflection of screening use. Indeed, a prior analysis^25^ found that PSA screening decreased after the USPSTF recommendation in 2012, and these reductions coincided, with decreased incidence of prostate cancer as shown in other data. However, unique to our data, we found that the incidence of prostate cancer began to decrease well before the 2012 recommendation, with the decrease appearing to more closely coincide with the 2008 recommendation. This recommendation was limited to men ages 75 and older. Thus, our data suggest 2 conclusions: that guidelines are associated with clinical care and that, in some respects, the adoption of guidelines may not have been as narrow as intended. For example, while men aged 75 years and older experienced the greatest (relative to other age groups) decrease in incidence beginning in 2005 (and theoretically associated with 2008 recommendations), men in all age groups had decreasing rates of prostate cancer diagnosis at approximately the same inflection point. This is contrary to what would be expected based on guideline statements alone.

Second, the understanding of differences in outcomes by race and ethnicity may elucidate underlying disparities in care delivery. For example, we found a temporal lag between when White men (2007) and African American and Hispanic men (2009) had a decrease in incidence relative to guideline changes. Some differences may be explained by systemic biases in our delivery of health care. It was previously established that there are disparities in incidence and mortality in African American men compared with White men^26^ but that social determinants of care may be associated with some observed differences.^27^ Further compounding the social determinants of care are findings that under a shared decision-making model, African American and White men note differences in the value of screening.^28^ Taken together, these data suggest that guidelines need to not only reflect evidence-based medicine, but also consider how complex the operationalization of the recommendations may be and support the need for diverse advocacy in the recommendation process.

### Limitations

This study has several limitations, such as not accounting for patient PSA screening history in the analysis. The Hispanic algorithm used in this study is required by SEER, NPCR, and the North American Association of Central Cancer Registries for all data submissions. It is possible that individuals may be misclassified as a result of the algorithm. A main function of US central cancer registries is to ascertain complete, timely, and accurate information on all reportable cancers in the US for the purpose of cancer surveillance, prevention, and control. Collection of additional information, such as cancer screening and access to primary care, is important but also cost prohibitive, time-consuming, and not a function that cancer registries can perform at this time without an increase in funding levels.

## Conclusions

This cross-sectional study was based on 14 years of all reported prostate cancer diagnoses in the US statewide central cancer registries and spanned 2 changes in PSA screening recommendations. It may provide important data on the timing and duration of changes in cancer diagnoses after changes in PSA screening recommendations and associated outcomes in cancer stages at diagnosis. Our findings may be of value to health professionals, patient navigators, and public health advocates to identify strategies to improve health education and shared decision-making among health professionals to reduce an increasing number of regional- and distant-staged cancers. Strategies tailored by social determinants of health and race may be beneficial.

## Notes

Supplement. **eFigure 1.** Join Point Trend Analysis of Localized Prostate Cancers by Ethnicity in the United States **eFigure 2.** Join Point Trend Analysis of Regional Prostate Cancer by Ethnicity in the United States **eFigure 3.** Join Point Trend Analysis of Distant Prostate Cancer by Ethnicity in the United States **eTable 1.** Total Prostate Cancer Count and Male Population From 2005 to 2018 by Age Group and Race **eTable 2.** Total Prostate Cancer Diagnosis Count and Male Population From 2005 to 2018 by Age Group and Ethnicity **eTable 3.** Annual Percent Change in Age-Adjusted Prostate Cancer Incidence Rates From 2005 to 2018 by Age Group and Ethnicity **eTable 4.** Annual Percent Change in Age-Adjusted Prostate Cancer Incidence Rates From 2005 to 2018 by Stage at Diagnosis and Ethnicity
