# Supplementary material for: Variations in Age-Adjusted Prostate Cancer Incidence Rates by Race and Ethnicity After Changes in Prostate-Specific Antigen Screening Recommendation
Source: JAMA Netw Open. 2022 Nov 7;5(11):e2240657. doi: 10.1001/jamanetworkopen.2022.40657 (PMC9641538; doi:10.1001/jamanetworkopen.2022.40657)
Supplement: Supplement. — eFigure 1. Join Point Trend Analysis of Localized Prostate Cancers by Ethnicity in the United States eFigure 2. Join Point Trend Analysis of Regional Prostate Cancer by Ethnicity in the United States eFigure 3. Join Point Trend Analysis of Distant Prostate Cancer by Ethnicity in the United States eTable 1. Total Prostate Cancer Count and Male Population From 2005 to 2018 by Age Group and Race eTable 2. Total Prostate Cancer Diagnosis Count and Male Population From 2005 to 2018 by Age Group and Ethnicity eTable 3. Annual Percent Change in Age-Adjusted Prostate Cancer Incidence Rates From 2005 to 2018 by Age Group and Ethnicity eTable 4. Annual Percent Change in Age-Adjusted Prostate Cancer Incidence Rates From 2005 to 2018 by Stage at Diagnosis and Ethnicity [file jamanetwopen-e2240657-s001.pdf]

Supplemental Online Content

Lai SM, Keighley J, Garimella S, Enko M, Parker WP. Variations in age-adjusted prostate cancer incidence rates by race and ethnicity after changes in prostate-specific antigen screening recommendation. *JAMA Netw Open.* 2022;5(11):e2240657. doi:10.1001/jamanetworkopen.2022.40657

eFigure 1. Join Point Trend Analysis of Localized Prostate Cancers by Ethnicity in the United States
eFigure 2. Join Point Trend Analysis of Regional Prostate Cancer by Ethnicity in the United States
eFigure 3. Join Point Trend Analysis of Distant Prostate Cancer by Ethnicity in the United States
eTable 1. Total Prostate Cancer Count and Male Population From 2005 to 2018 by Age Group and Race
eTable 2. Total Prostate Cancer Diagnosis Count and Male Population From 2005 to 2018 by Age Group and Ethnicity
eTable 3. Annual Percent Change in Age-Adjusted Prostate Cancer Incidence Rates From 2005 to 2018 by Age Group and Ethnicity
eTable 4. Annual Percent Change in Age-Adjusted Prostate Cancer Incidence Rates From 2005 to 2018 by Stage at Diagnosis and Ethnicity

This supplemental material has been provided by the authors to give readers additional information about their work.

© 2022 Lai SM et al. *JAMA Network Open.*

eFigure 1. Joinpoint Trend Analysis of Localized Prostate Cancers by Ethnicity in the United States

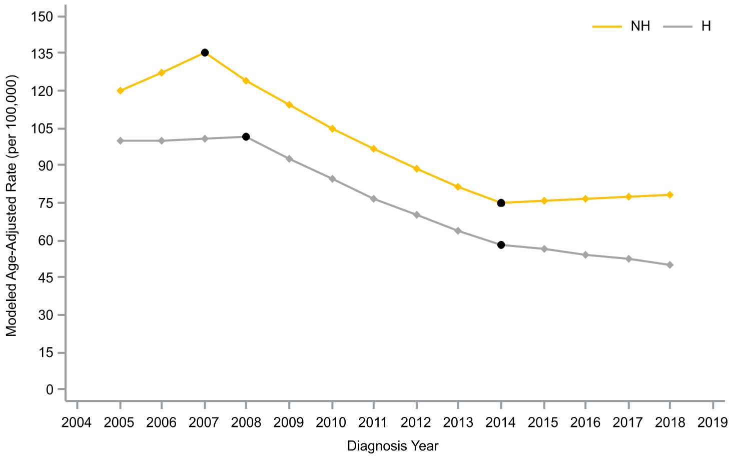

*** black dot indicates Joinpoint location**
*** H, Hispanic; NH, Non-Hispanic;**

© 2022 Lai SM et al. *JAMA Network Open.*

eFigure 2. Joinpoint Trend Analysis of Regional Prostate Cancer by Ethnicity in the United States

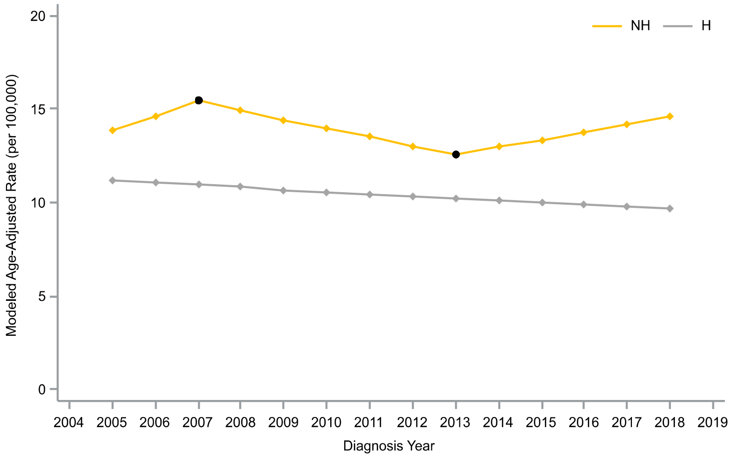

*** black dot indicates Joinpoint location**
*** H, Hispanic; NH, Non-Hispanic;**

© 2022 Lai SM et al. *JAMA Network Open.*

eFigure 3. Joinpoint Trend Analysis of Distant Prostate Cancer by Ethnicity in the United States

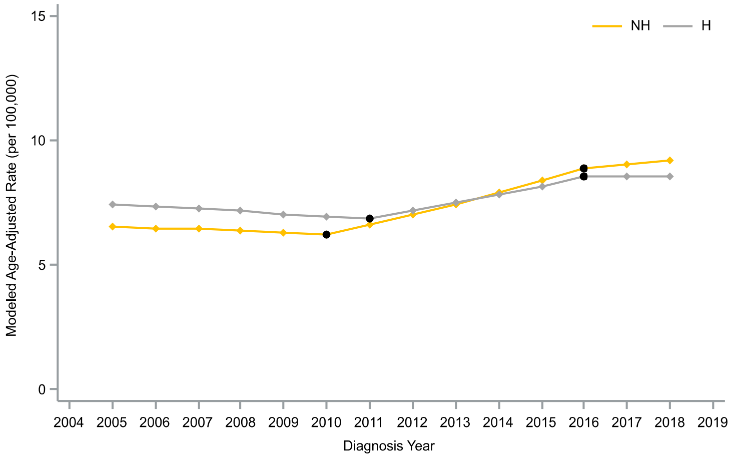

*** black dot indicates Joinpoint location**
*** H, Hispanic; NH, Non-Hispanic;**

© 2022 Lai SM et al. *JAMA Network Open.*

eTable 1. Total Prostate Cancer Count and Male Population From 2005 to 2018 by Age Group and Race

Diagnosis Year	Race	Under 65 years		65 - 74 years		75+ years	
		Total Cases	Total Population	Total Cases	Total Population	Total Cases	Total Population
**2005**	AA	13,647	17,509,711	9,652	765,660	5,132	454,328
	AI/AN	347	1,689,270	283	57,803	155	28,638
	API	1,150	6,521,030	1,481	313,668	1,076	188,295
	White	63,072	104,029,128	61,667	7,545,997	45,268	6,093,550
**2006**	AA	15,123	17,769,164	10,081	785,597	5,103	461,878
	AI/AN	372	1,753,538	293	60,869	179	29,983
	API	1,329	6,741,337	1,652	332,241	1,140	198,025
	White	71,264	104,649,981	67,659	7,673,682	47,236	6,190,970
**2007**	AA	16,667	18,030,125	10,784	807,080	5,323	471,475
	AI/AN	386	1,821,969	353	64,441	188	31,539
	API	1,501	6,952,475	1,836	351,839	1,151	208,139
	White	77,765	105,171,176	71,228	7,879,565	47,681	6,275,031
**2008**	AA	16,987	18,284,415	10,714	835,548	4,924	482,980
	AI/AN	382	1,895,467	333	68,783	179	33,126
	API	1,598	7,168,590	1,726	371,854	1,048	219,352
	White	75,249	105,557,571	68,373	8,225,250	42,740	6,347,015
**2009**	AA	17,913	18,529,766	11,016	863,857	4,749	494,311
	AI/AN	444	1,970,761	329	73,152	185	34,906
	API	1,575	7,371,064	1,807	393,485	1,063	230,828
	White	75,415	105,910,439	67,045	8,527,448	38,079	6,407,437
**2010**	AA	17,588	18,766,009	10,685	892,938	4,498	511,248
	AI/AN	422	2,033,111	359	77,297	172	36,937
	API	1,597	7,567,308	1,768	415,241	995	244,770
	White	71,763	106,238,989	65,374	8,782,624	36,210	6,510,546
**2011**	AA	18,488	18,973,621	10,920	922,635	4,451	530,588
	AI/AN	401	2,054,687	344	81,453	193	39,318
	API	1,742	7,771,898	1,913	439,480	1,102	262,678
	White	74,131	106,483,756	67,517	9,032,970	35,736	6,618,741
**2012**	AA	16,621	19,163,066	10,179	979,528	3,890	550,227
	AI/AN	399	2,074,738	337	88,660	149	41,901
	API	1,513	7,978,230	1,719	470,877	889	282,255
	White	61,486	106,362,719	58,577	9,665,982	29,804	6,738,100
**2013**	AA	16,351	19,342,455	10,097	1,037,258	3,931	569,557
	AI/AN	390	2,094,568	339	94,944	151	44,723
	API	1,478	8,189,768	1,782	504,154	926	302,859
	White	58,309	106,320,729	58,937	10,143,966	29,388	6,869,366
**2014**	AA	16,218	19,522,616	10,077	1,097,878	3,629	591,343

© 2022 Lai SM et al. *JAMA Network Open.*

	AI/AN	353	2,114,180	320	101,686	144	47,704
	API	1,364	8,413,442	1,666	539,717	866	324,956
	White	55,720	106,333,645	57,383	10,588,689	28,975	7,021,786
**2015**	AA	16,815	19,700,913	11,023	1,162,920	3,918	615,318
	AI/AN	403	2,134,164	335	108,352	154	51,096
	API	1,531	8,647,800	2,024	577,922	1,016	349,388
	White	57,105	106,383,509	62,987	11,014,823	31,015	7,164,570
**2016**	AA	17,077	19,873,636	11,820	1,227,593	4,027	638,805
	AI/AN	343	2,152,856	353	114,886	178	54,643
	API	1,595	8,879,948	1981	611,481	1,145	373,298
	White	57,439	106,394,083	65,763	11,426,540	32,442	7,340,333
**2017**	AA	17,637	20,031,123	12,331	1,285,699	4,176	663,947
	AI/AN	426	2,169,960	368	121,107	191	58,373
	API	1,688	9,087,008	2,286	645,384	1,252	398,415
	White	59,814	106,302,520	71,314	11,800,803	33,974	7,561,291
**2018**	AA	17,063	20,179,107	12,443	1,343,795	4,083	694,342
	AI/AN	364	2,185,693	343	127,780	146	62,517
	API	1,587	9,284,297	2,330	681,357	1,271	424,615
	White	57,927	106,172,764	71,020	12,093,153	34,561	7,879,259

Surveillance, Epidemiology, and End Results (SEER) Program (www.seer.cancer.gov) SEER*Stat Database: Populations – Total U.S. (1990-2018) – Linked to County Attributes – Total U.S., 1969-2018 Counties, National Cancer Institute, DCCPS, Surveillance Research Program, released December 2019.

Abbreviations: AA, African American; AI/AN, American Indian or Alaska Native; API, Asian Pacific Islander

© 2022 Lai SM et al. *JAMA Network Open.*

eTable 2. Total Prostate Cancer Diagnosis Count and Male Population From 2005 to 2018 by Age Group and Ethnicity

Diagnosis Year	Ethnicity	Under 65 Years		65 - 74 Years		75+ Years	
		Total Cases	Total Population	Total Cases	Total Population	Total Cases	Total Population
2005	Hispanic	4,440	21,052,837	4,490	583,926	2,812	349,143
	Non-Hispanic	75,068	108,696,302	69,579	8,099,202	49,467	6,415,668
2006	Hispanic	4,955	21,787,543	4,855	608,408	2,906	368,726
	Non-Hispanic	84,743	109,126,477	76,077	8,243,981	51,535	6,512,130
2007	Hispanic	5,490	22,518,195	5,141	635,683	3,124	388,185
	Non-Hispanic	92,825	109,457,550	80,720	8,467,242	52,267	6,597,999
2008	Hispanic	5,749	23,243,862	5,125	669,611	3,136	407,421
	Non-Hispanic	90,415	109,662,181	77,619	8,831,824	46,628	6,675,052
2009	Hispanic	6,143	23,927,997	5,474	705,481	2,882	428,545
	Non-Hispanic	91,398	109,854,033	76,609	9,152,461	42,074	6,738,937
2010	Hispanic	5,865	24,547,221	5,193	744,031	2,800	453,518
	Non-Hispanic	87,939	110,058,196	75,118	9,424,069	40,120	6,849,983
2011	Hispanic	6,080	25,019,421	5,335	784,984	2,667	479,349
	Non-Hispanic	91,230	110,264,541	77,610	9,691,554	39,875	6,971,976
2012	Hispanic	5,618	25,464,791	4,973	839,834	2,454	505,732
	Non-Hispanic	76,453	110,113,962	67,813	10,365,213	33,169	7,106,751
2013	Hispanic	5,587	25,906,736	5,067	896,579	2,572	533,181
	Non-Hispanic	72,940	110,040,784	68,079	10,883,743	32,697	7,253,324
2014	Hispanic	5,588	26,370,961	4,844	954,539	2,436	562,588
	Non-Hispanic	70,070	110,012,922	66,370	11,373,431	31,987	7,423,201
2015	Hispanic	5,908	26,869,883	5,174	1,018,511	2,559	593,849
	Non-Hispanic	72,315	109,996,503	73,522	11,845,506	34,731	7,586,523
2016	Hispanic	6,023	27,374,454	5,436	1,086,128	2,792	625,427
	Non-Hispanic	73,164	109,926,069	77,502	12,294,372	36,672	7,781,652
2017	Hispanic	6,182	27,839,138	6,005	1,153,056	2,852	658,663
	Non-Hispanic	76,836	109,751,473	83,994	12,699,937	38,532	8,023,363
2018	Hispanic	6,159	28,310,011	5,832	1,224,439	2,810	696,563
	Non-Hispanic	74,275	109,511,850	83,845	13,021,646	38,972	8,364,170

National Program of Cancer Registries and Surveillance, Epidemiology, and End Results (SEER) Program SEER*Stat Database: NPCR and SEER Incidence – U.S. Cancer Statistics Public Use Research Database, 2020 Submission (2001-2018). United States Department of Health and Human Services. Centers of Disease Control and Prevention and National Cancer Institute. Released June 2021. Accessed at www.cdc.gov/cancer/uscs/public-use.

eTable 3. Annual Percent Change in Age-Adjusted Prostate Cancer Incidence Rates From 2005 to 2018 by Age Group and Ethnicity

**Hispanic**				
**Age Groups**	**Number of Joinpoints**	**Time Segment**	**APC[1] (95% C.I.)**	**P value**
Under 65 Years	2	2005-2009	1.11 (-1.39 to 3.68)	0.32
		2009-2013	-7.78 (-11.15 to -4.28)	0.002
		2013-2018	-2.05 (-3.63 to -0.45)	0.02
65 – 74 Years	2	2005-2009	-0.49 (-4.30 to 3.47)	0.77
		2009-2014	-7.92 (-11.36 to -4.35)	0.002
		2014-2018	-0.82 (-4.39 to 2.88)	0.60
75+ Years	2	2005-2008	-1.37 (-6.51 to 4.06)	0.55
		2008-2012	-10.81 (-15.56 to -5.79)	0.002
		2012-2018	-2.51 (-4.32 to -0.66)	0.02
**Non-Hispanic**				
Under 65 Years	2	2005-2007	8.73 (-4.99 to 24.43)	0.18
		2007-2014	-5.72 (-8.07 to -3.32)	0.001
		2014-2018	0.50 (-5.02 to 6.35)	0.84
65 – 74 Years	2	2005-2007	5.64 (-9.81 to 23.75)	0.43
		2007-2014	-6.64 (-9.10 to -4.13)	0.001
		2014-2018	2.68 (-2.15 to 7.75)	0.23
75+ Years	2	2005-2007	0.53 (-10.93 to 13.47)	0.92
		2007-2013	-8.92 (-11.62 to -6.13)	<0.001
		2013-2018	1.45 (-1.73 to 4.73)	0.31

[1]Annual Percent Change

© 2022 Lai SM et al. *JAMA Network Open.*

eTable 4. Annual Percent Change in Age-Adjusted Prostate Cancer Incidence Rates From 2005 to 2018 by Stage at Diagnosis and Ethnicity

**Stage at diagnosis[1]: Localized**				
**Ethnicity**	**Number of Joinpoints**	**Time Segment**	**APC[2] (95% C.I.)**	**P value**
Hispanic	2	2005-2008	0.56 (-4.33 to 5.70)	0.79
		2008-2014	-8.80 (-10.69 to -6.87)	<0.001
		2014-2018	-3.61 (-6.45 to -0.69)	0.02
Non-Hispanic	2	2005-2007	6.11 (-8.06 to 22.45)	0.35
		2007-2014	-8.17 (-10.43 to -5.85)	<0.001
		2014-2018	1.16 (-3.64 to 6.20)	0.58
**Stage at diagnosis[1]: Regional**				
Hispanic	0	2005-2018	-1.07 (-1.76 to -0.37)	0.006
Non-Hispanic	2	2005-2007	5.49 (-7.97 to 20.92)	0.38
		2007-2013	-3.32 (-6.35 to -0.18)	0.04
		2013-2018	2.92 (-0.35 to 6.29)	0.07
**Stage at diagnosis[1]: Distant**				
Hispanic	2	2005-2011	-1.46 (-3.18 to 0.30)	0.09
		2011-2016	4.60 (1.36 to 7.94)	0.01
		2016-2018	-0.09 (-7.29 to 7.66)	0.98
Non-Hispanic	2	2005-2010	-1.03 (-2.11 to 0.06)	0.06
		2010-2016	6.08 (5.08 to 7.09)	<0.001
		2016-2018	1.76 (-1.67 to 5.32)	0.26

[1]SEER Summary Stage at diagnosis; [2]Annual Percent Change

© 2022 Lai SM et al. *JAMA Network Open.*
